# Tunable Broadband Nonlinear Optical Properties of Black Phosphorus Quantum Dots for Femtosecond Laser Pulses

**DOI:** 10.3390/ma10020210

**Published:** 2017-02-21

**Authors:** Xiao-Fang Jiang, Zhikai Zeng, Shuang Li, Zhinan Guo, Han Zhang, Fei Huang, Qing-Hua Xu

**Affiliations:** 1 State Key Laboratory of Luminescent Materials and Devices, Institute of Polymer Optoelectronic Materials and Devices, South China University of Technology, Guangzhou 510640, China; msxfjiang@scut.edu.cn (X.-F.J.); zeng.zhikai@mail.scut.edu.cn (Z.Z.); mssli@scut.edu.cn (S.L.); 2 SZU-NUS Collaborative Innovation Center for Optoelectronic Science and Technology, and Key Laboratory of Optoelectronic Devices and Systems of Ministry of Education and Guangdong Province, Shenzhen University, Shenzhen 518060, China; guozhinan@126.com (Z.G.); hzhang@szu.edu.cn (H.Z.); 3 National University of Singapore (Suzhou) Research Institute, Suzhou 215123, China; 4 Department of Chemistry, National University of Singapore, Singapore 117543, Singapore

**Keywords:** black phosphorus, quantum dots, nonlinear optics, two-photon absorption, femtosecond laser

## Abstract

Broadband nonlinear optical properties from 500 to 1550 nm of ultrasmall black phosphorus quantum dots (BPQDs) have been extensively investigated by using the open-aperture Z-scan technique. Our results show that BPQDs exhibit significant nonlinear absorption in the visible range, but saturable absorption in the near-infrared range under femtosecond excitation. The calculated nonlinear absorption coefficients were found to be (7.49 ± 0.23) × 10^−3^, (1.68 ± 0.078) × 10^−3^ and (0.81 ± 0.03) × 10^−3^ cm/GW for 500, 700 and 900 nm, respectively. Femtosecond pump-probe measurements performed on BPQDs revealed that two-photon absorption is responsible for the observed nonlinear absorption. The saturable absorption behaviors observed at 1050, 1350 and 1550 nm are due to ground-state bleaching induced by photo-excitation. Our results suggest that BPQDs have great potential in applications as broadband optical limiters in the visible range or saturable absorbers in the near-infrared range for ultrafast laser pulses. These ultrasmall BPQDs are potentially useful as broadband optical elements in ultrafast photonics devices.

## 1. Introduction

The upsurge of two-dimensional (2D) materials was triggered by the discovery of graphene in 2004 when it was isolated from its parent graphite [1]. Due to their outstanding physical and chemical properties and promising applications in various fields such as photovoltaics [2,3] and electronics [4,5], 2D materials became a new class of nanomaterials that have a groundbreaking impact on nanotechnology. Current studies on 2D materials mainly focus on graphene and wide-bandgap transitional metal dichalcogenides (TMDs) such as molybdenum disulfide (MoS_2_) [6]. Most importantly, there is significant interest in studying 2D materials’ optical properties and their applications as nonlinear optical materials (optical limiters and saturable absorbers). Graphene and graphene oxide have been reported to display broadband optical-limiting properties due to strong two-photon absorption [7,8,9,10]. Broadband nonlinear absorption was also discovered in TMDs [11,12] and their applications in ultrafast lasers have been investigated [13,14]. Besides 2D materials, ultrasmall quantum dots (QDs) are being also extensively studied due to their unique electronic and optical properties arising from the quantum confinement effect [15].

Black phosphorous (BP), also known as phosphorene, is the latest member of the family of 2D materials [16,17]. BP is the most thermodynamically stable allotrope of phosphorus [18] and its unique structure as well as fascinating optical and electronic properties have attracted a lot of research interests [19]. As bulk BP consists of phosphorene monolayers stacked together by van der Waals force, it can be mechanically [20] or chemically exfoliated [21,22,23] into few-layer or single-layer nanosheets, or quantum dots. Due to its attractive properties, BP has been applied to field-effect transistors (FET) [17,24]. Few-layer BP can be used in thin-film solar cells [25] and p-n diodes [26]. It is also theoretically predicted to be used in flexible ambipolar transistors [27], energy storage [28] and moveable vibratory devices [29]. In particular, it has been previously reported that few-layer BP can act as an effective saturable absorber for ultrashort pulse generation in solid-state and fiber mode–locked lasers operating in the 1000–2000 nm wavelength range [30,31,32,33,34,35].

Unlike graphene’s zero-bandgap nature which limits its electronic and photonic applications, it is noteworthy that the bandgap of BP depends on the number of layers, ranging from 0.3 (bulk) to 2.0 eV (single layer) [36,37]. It has also been found that the applied strain force [38,39], stacking order [25] and external electric field [40] can also modulate the bandgap of BP. The tunable bandgap of BP shows great potential in bridging the space between zero-bandgap semi-metallic graphene and wide-bandgap TMDs (1–2 eV). Hence, BP is considered to be suitable for extremely broadband nonlinear optical applications, including optical limiters and saturable absorbers [30,31,32,33,34,35]. The past decades have witnessed significant research efforts in developing broadband optical materials. Optical-limiting materials exhibit decreased transmittance at high-input laser intensity, which can be used to protect human eyes and sensitive instruments from damage by high-intensity laser beams [41]. In contrast, saturable absorbers show an increased transmittance at high-input laser intensity, which can be utilized in pulse compression, mode locking and Q-switching [42].

Herein, for the first time, black phosphorus quantum dots (BPQDs) are extensively investigated in broadband femtosecond nonlinear optical properties from visible to near-infrared (near-IR) range. Based on the simple solution exfoliation method, a suspension of BPQDs was prepared in N-Methylpyrrolidone solvent (NMP). The utrasmall BPQDs were experimentally demonstrated to display nonlinear optical responses in a broad wavelength range by femtosecond open-aperture Z-scan measurements. Under femtosecond laser excitation, BPQDs exhibited significant nonlinear absorption in the visible range, but saturable absorption in the near-infrared (near-IR) range. Femtosecond pump-probe measurements performed on BPQDs revealed that two-photon absorption is primarily responsible for the observed nonlinear absorption. The results suggest that BPQDs have great potential in applications as broadband optical limiters in the visible range or as saturable absorbers in the near-IR range for ultrafast laser pulses. These ultrasmall BPQDs are potentially useful as broadband optical elements in fiber lasers and other ultrafast photonics devices.

## 2. Results and Discussion

### 2.1. Sample Preparation and Characterizations

**Figure 1:**
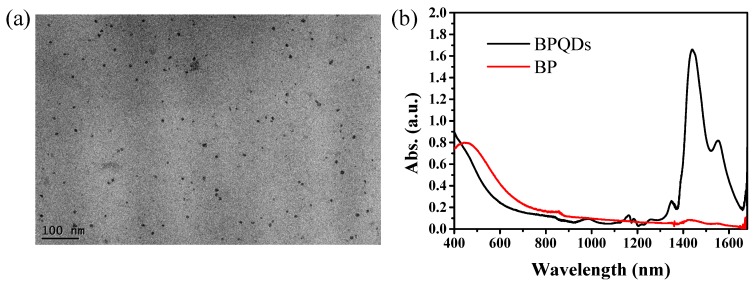
(**a**) TEM image of BPQDs; (**b**) absorption spectra of BP and BPQDs.

Mechanical and liquid exfoliation methods are commonly used as simple and effective techniques to prepare 2D nanomaterials and QDs from bulk crystals, such as BP [21,22] and graphene [43]. In the simple liquid exfoliation technique, solvents with a suitable surface energy can serve as stable dispersions for layered materials. Here in this article, the solvent exfoliation combined with probe sonication and bath sonication were used to fabricate BPQDs dispersed in N-Methylpyrrolidone (NMP) solvent [44]. BP has been known to be sensitive to water and oxygen and can be oxidized under visible-light irradiation [45,46]. In our experiments, BPQDs were prepared in ambient conditions and dispersed in NMP solution to avoid any exposure to air as much as possible. The absorption and Raman spectra were measured and compared before and after nonlinear optical property measurements to check for any possible degradation. More experimental details can be found in Section 3. Transmission electron microscopy (TEM) measurements were conducted to examine the morphology of the as-prepared BPQDs. The TEM image (Figure 1a) shows that the ultrasmall BPQDs have an average lateral size of about 2–3 nm, which corresponds to the stacking number of layers of 2 ± 1. The absorption spectra (Figure 1b) show that BPQDs have a broad absorption band, spanning from the UV to near-IR range. The absorption band at the UV-visible range is similar to those of other 2D layered materials such as graphene oxide, as well as few-layer BP. Compared to BP, a huge absorption band in the near-IR range from 1250 to 1630 nm with two absorption maxima at 1438 and 1550 nm was observed in the BPQD dispersion. This near-IR broad absorption band might be due to the defect state absorption induced by the ultrasmall size of the QDs [47], which indicates possible interesting nonlinear optical properties of BPQDs both in visible and near-IR regions.

**Figure 2:**
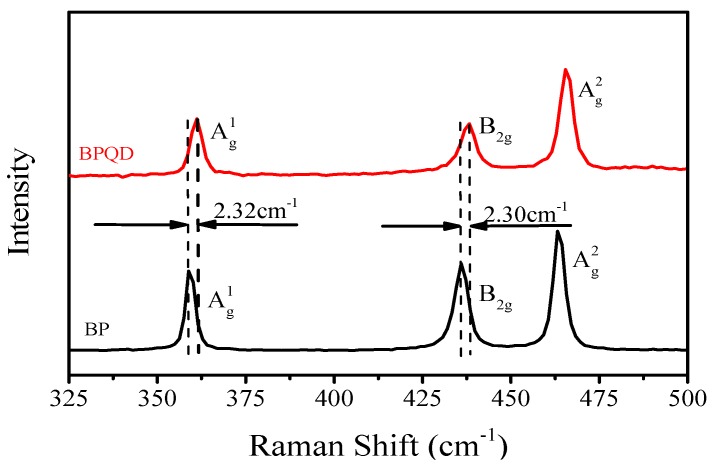
Raman spectra of BPQDs and bulk BP.

BPQDs were also characterized by Raman spectroscopy. Samples were prepared by spin-coating the BPQD dispersion onto the quartz substrates, drying them on a heating plate under 348 K for 5 min, and then keeping them in N_2_ for the next 6 h. As shown in Figure 2, the three observed peaks can be attributed to one out-of-plane phonon mode ($A_{g}^{1}$) at 361.16 cm^−1^, and two in-plane modes, which are B_2g_ and $A_{g}^{2}$, at 438.22, and 465.65 cm^−1^, respectively, consistent with the reported values [15]. Compared to the bulk BP, both B_2g_ and $A_{g}^{2}$ modes of BPQDs are red-shifted by 2.30 cm^−1^, while the $A_{g}^{1}$ mode is red-shifted by 2.32 cm^−1^. It can be explained that the Raman shift is dependent on thickness and lateral dimensions. The frequency difference between the $A_{g}^{1}$ and $A_{g}^{2}$ modes of BPQDs equals 104.3 cm^−1^, which is larger than the reported value of bulk BP [48] and confirms that we successfully reduced the thickness of the BP.

### 2.2. The Nonlinar Optical Properties of BPQDs in Visible Range

**Figure 3:**
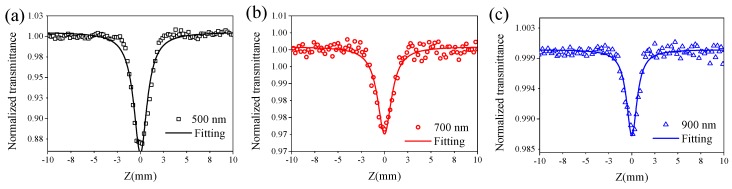
The open aperture Z-scan results of BPQD dispersion in NMP measured by femtosecond laser pulses with central wavelength at 500 nm (**a**); 700 nm (**b**) and 900 nm (**c**) under the same peak power intensity of 147 GW/cm^2^, respectively.

Open-aperture femtosecond Z-scan measurements were used to characterize the nonlinear optical properties of BPQDs. The BPQD dispersion was placed in a 1 mm cuvette for Z-scan measurements. The detailed experimental setup is described in Section 3. Briefly, femtosecond laser pulses with a pulse duration of 120 fs and a repetition rate of 1 kHz were focused onto the cuvette with the samples, which were moved towards and away from the focus by using a motorized translational stage to study the excitation power intensity–dependent transmission of samples. Similar Z-scan measurements were performed with femtosecond laser pulses at 500, 700 and 900 nm, which were generated by an optical parametric amplifier (TOPAS-Prime) pumped by a mode-locked Ti:sapphire oscillator–seeded regenerative amplifier. The Z-scan results showed that a very weak saturable absorption effect was exhibited in the visible spectra range upon low excitation intensity due to ground-state bleaching. As the excitation intensity increased, reverse saturable absorption occurred and finally became dominant. The results under the high excitation peak power intensity of 147 GW/cm^2^ are shown in Figure 3. The subfigures (a–c) are corresponding to the Z-scan results at 500 nm (a), 700 nm (b) and 900 nm (c), respectively. NMP solvent in a 1 mm cuvette was measured and no nonlinear absorption was found, which excludes the effects of NMP and the 1 mm cuvette. For all wavelengths, the normalized transmittance gradually decreased as the sample move towards the focus point (Z = 0), indicating an optically induced reverse saturable absorption. The Z-scan curves can be fitted to achieve the nonlinear absorption coefficient β with the following approximate equation:

$$T(Z)=\sum_{m=0}^{\infty }{\left(-\beta I_{0}L_{eff}\right)}^{m}/{\left(1+Z^{2}/Z_{0}^{2}\right)}^{m}{\left(m+1\right)}^{3/2}\approx 1-\beta I_{0}L_{eff}/2^{3/2}(1+Z^{2}/Z_{0}^{2}),$$

where Z is the distance between the sample and the focus; $Z_{0}$ is the Rayleigh diffraction length; $I_{0}$ is the peak power density of the excitation laser pulses; $L_{eff}={(1-e}^{-\alpha_{0}L})/\alpha_{0}$ is the effective sample length; L is the sample length; $\alpha_{0}$ is the linear absorption coefficient. The fitting values of nonlinear absorption coefficient β at 500, 700 and 900 nm were found to be (7.49 ± 0.23) × 10^−3^, (1.68 ± 0.078) × 10^−3^ and (0.81 ± 0.03) × 10^−3^ cm/GW for BPQDs, respectively. The obtained nonlinear absorption coefficients of these BPQDs are higher than those of other optical-limiting materials such as CdSe QDs with average size of around 2 nm ((1.1 ± 0.15) × 10^−3^ cm/GW) [49], CdO nanoflakes (0.69 × 10^−3^ cm/GW) [50] and Fe_2_O_3_ hexagonal nanostructures (0.82 × 10^−3^ cm/GW) [51], obtained by using the similar femtosecond laser pulses. These results indicated that BPQDs can act as good broadband optical-limiting materials in the visible range. The results are similar to the previous studies on the nonlinear absorption properties of BP nanoplatelets at 800 nm [52]. These nonlinear absorption properties can be well analyzed based on the band structure of BP. The bandgap of bulk BP is ~0.3 eV, and the bandgap increases with the layer decreasing. With the size and layer of BPs decreasing, the quantum effects become increasingly important. Upon excitation by the photon with an energy of 1.3–2.48 eV (equivalent to 500–900 nm), electrons cannot be promoted from the top of the valence band (VB) through direct transition but can only jump by indirect transition or through the defects level, as the photon energy is much larger than the bandgap of BP. For the energy (2.6–4.96 eV) of two photons at 500–900 nm, when the excitation intensity is high, electrons can be directly promoted from the top of the valence band through the two-photon absorption (TPA) process, which deduced the observed significant optical-limiting properties.

### 2.3. The Ultrafast Carrier Dynamics of BPQDs

**Figure 4:**
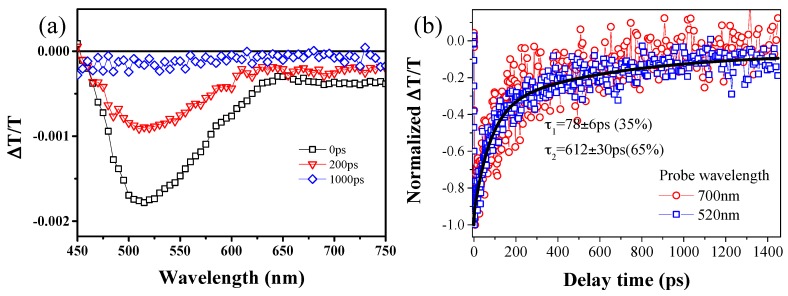
(**a**) Transient absorption spectra of BPQDs in NMP at the different delay times under excitation at 400 nm; (**b**) Single-wavelength dynamics at probed at 525 and 700 nm, measured by femtosecond pump-probe technique.

To better understand the possible mechanisms behind the nonlinear absorption properties in the BPQDs, transient absorption (TA) spectra as well as the single-wavelength dynamics were measured under excitation at 400 nm (3.1 eV) with a pump fluence of 16 μJ/cm^2^. TA spectra of BPQDs in NMP at various delay times are shown in Figure 4a. A broad negative transient differential transmission band spanning from 450 to 750 nm was found. The observed negative transmission change signal is commonly assigned to the excited state absorption. This result is also consistent with the previously reported photon-induced free carrier participated interband transient absorption in few-layer BP and graphene [53,54]. As mentioned above, the excitation photon energy (3.1 eV) is much larger than the bandgap of BP. Thus, the negative transmission change signal induced by interband transient absorption could be attributed to the multiphoton absorption process. Our pump-probe results further support the nonlinear absorption behaviors observed by the Z-scan measurement in the visible range. Figure 4b shows the single-wavelength dynamics probed at the wavelengths of 520 and 700 nm. The photo-induced absorption decays probed at different wavelengths show no difference and could be well fit with a bi-exponential equation with time constants of τ_1_ = 78 ± 6 ps (35%) and τ_2_ = 612 ± 30 ps (65%). The two characteristic time constants on the order of tens of picoseconds and hundreds of picoseconds could be attributed to the electron-phonon and slower phonon-phonon scattering during the interband transition.

### 2.4. The Nonlinar Optical Properties of BPQDs in Near-IR Range

**Figure 5:**
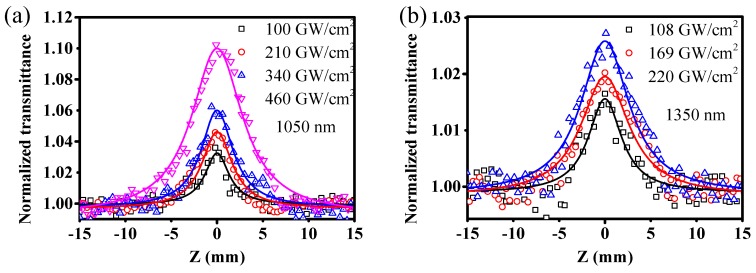
The open aperture Z-scan results of BPQDs at 1050 nm (**a**) and 1350 nm (**b**) under different excitation peak power intensities at the focal point.

We also investigated the nonlinear absorption responses of BPQDs in NMP at 1050, 1350 nm, respectively. The open aperture Z-scan measurement results under different excitation peak power intensities at the focal point are shown in Figure 5. The normalized transmittance gradually increased with the approaching of the BPQD sample with respect to the focal point (Z = 0), indicating that the absorption of BPQDs becomes saturated with the increase of the incident pump intensity. This is well known as the saturable absorption behavior. As shown in the absorption spectra, there is a strong absorption band in the near-IR range. The observed saturable absorption activity can be ascribed to the ground-state bleaching induced by photo-excitation. Figure 5 shows the power-dependent saturable absorption. It can be seen that the peaks of the open Z-scan curves increased with the increasing input power intensity. These results further confirm that the saturable absorption responses indeed originate from the intrinsic optical absorption effects in BPQDs other than from artifacts such as sample damage or contamination. When the excitation intensity increased further until it reached the photo-damage threshold, the reverse saturable absorption effect was not observed. These results can be ascribed to smaller two-photon absorption coefficients of BPQDs at the near-IR spectra range, which are not enough to overcome the ground-state bleaching. These results are consistent with the wavelength dependence of the two-photon absorption coefficient in the visible spectra range: the two-photon absorption coefficient decreased with the increasing wavelength.

**Figure 6:**
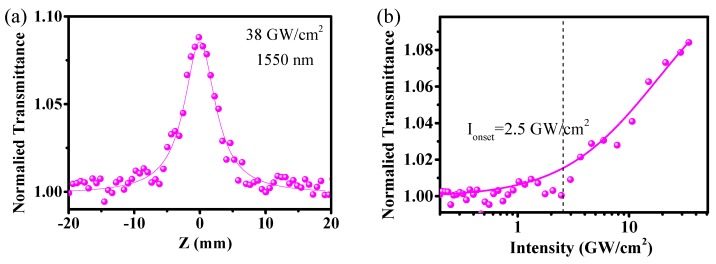
The open aperture Z-scan results of BPQDs at 1550 nm (**a**) and its corresponding saturable absorption curve (**b**).

Similar measurements have also been performed at 1550 nm. Figure 6 shows the Z-scan curve of BPQDs under the excitation peak power intensity of 38 GW/cm^2^ and the corresponding fitting curve of saturable absorption. Based on the relation between the laser beam spot size and the relative separation, the nonlinear saturable absorption curve could be derived (Figure 6b). The onset of saturation intensity was around 2.5 GW/cm^2^ and the modulation depth was 8.2% at 1550 nm. Therefore, the measured results indicate that the as-prepared BPQDs can be used as a saturable absorber for ultrafast laser pulse generation in near-IR fiber lasers.

## 3. Materials and Methods

### 3.1. Synthesis of BPQDs

NMP was used to isolate the BPQDs from the bulk BP crystal. First, the BP bulk crystal (99.998%, purchased from smart elements) was pulverized to prepare the BP powder. The dispersed suspension of BPQDs was then prepared by ultrasound probe sonication followed by ice bath sonication of bulk BP powder in 1-methyl-2-pyrro-lidone (NMP, 99.5% anhydrous, purchased from Aladdin Reagents). Then 25 mg of the BP powder was dispersed into 25 mL of NMP in a 50 mL sealed conical tube and sonicated with a sonic tip for 3 h at the power of 1200 W. The ultrasonic frequency ranged from 19 to 25 kHz and the ultrasound probe worked 2 s with an interval of 4 s. Afterwards, an ultrasonic bath was adopted to sonicate the dispersion consecutively for another 10 h at the power of 300 W. During the sonication, the temperature of the sample solution was kept and monitored below 277 K in an ice bath. The dispersion was then centrifuged at the speed of 7000 rpm for 20 min. The supernatant containing BPQDs was collected. To further increase the concentration of the BPQDs dispersion, the collected solution was centrifuged for 20 min at the speed of 12,000 rpm. The resulting supernatant was collected and was kept in a sealed tube for further tests.

Transmission electron microscopy (TEM) measurements were conducted to examine the morphology of the as-prepared BPQDs. The TEM images were taken on the Tecnai G2 F20 S-Twin transmission electron microscope at an acceleration voltage of 200 kV. The absorption spectra were taken by a Shimadzu UV3600 spectroscopy with QS-grade quartz cuvette at room temperature.

### 3.2. Nonlinear Optical Property Measured by Z-Scan Technique

The open aperture Z-scan measurements were performed by using an optical parametric amplifier (TOPAS-Prime) pumped by a mode-locked Ti:sapphire oscillator seeded regenerative amplifier (Spectra-Physics Spitfire Ace), which gives output laser pulses with tunable central wavelength from UV to near-IR range, pulse duration of ~120 fs and repetition rate of 1 kHz. The laser beam was focused onto the sample with a beam radius of ~23 μm. Samples were prepared in the quartz cuvette with the optical path length of 1 mm. The transmittance of the samples was measured as a function of input intensity, which was varied by moving the samples in and out of beam focus along the z-axis. At least five cycles were repeated to reduce the experimental error and the average was calculated to plot the Z-scan curves.

### 3.3. Pump-Probe and Transient Absorption Spectra Mansurement

**Figure 7:**
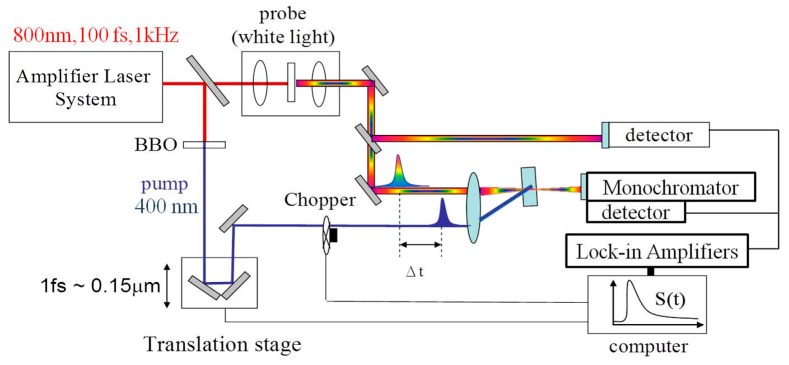
Experimental setup of pump-probe and transient absorption measurements.

In our experiments, a Ti:sapphire oscillator seeded regenerative amplifier laser system (Spectra Physics Spitfire Ace) with output pulse energy of 2 mJ at 800 nm and a repetition rate of 1 kHz was used as the source of the pump and beam pulses. The detailed experimental setup is described in Figure 7. The 800 nm laser beam was split into two portions. One portion passes through a BBO crystal to generate the 400 nm pump beam by second harmonic generation. The other portion of the 800 nm beam was used to generate white light continuum in a 2.5 mm sapphire plate. The white light beam was split into two portions: one as the probe and another as the reference. The pump beam was focused onto the sample with a beam size with 300 µm in diameter and overlapped with the smaller probe beam (100 µm in diameter). The probe beam is collected after passing through the sample and its intensity is monitored by a photodiode as the detector. Both the detectors of probe and reference beam are connected to the lock-in amplifier to correct the pulse-to-pulse intensity fluctuations. The time delay between the pump and probe pulse was varied by a computer-controlled translation stage. The pump beam was modulated by an optical chopper at the frequency of 500 Hz. In a single-wavelength dynamics scan, a fixed probe wavelength is picked and the transmittance change of probe with pump and without pump (∆T/T) was monitored as a function of the various delay between pump and probe beam. The transient absorption spectra at different delay time were measured by passing the probe through a monochromator before detector.

## 4. Conclusions

In conclusion, BPQDs were fabricated by a simple solvent exfoliation method. The nonlinear absorption properties were investigated with Z-scan measurements using femtosecond laser pulses at 500, 700, 900, 1050, 1350 and 1550 nm. These ultrasmall BPQS in NMP suspension exhibited broadband optical-limiting activities in the visible range. The nonlinear absorption coefficient was found to be (7.49 ± 0.23) × 10^−3^, (1.68 ± 0.078) × 10^−3^ and (0.81 ± 0.03) × 10^−3^ cm/GW for 500, 700 and 900 nm, respectively. Femtosecond pump-probe measurements performed on the BPQD suspension further support that nonlinear absorption due to two-photon absorption played an important role in the observed optical-limiting activities of the BPQDs. Furthermore, saturable absorption due to ground-state bleaching was found in BPQDs by photo-excitation at 1050, 1350 and 1550 nm. Our results will provide the basis for applications of BPQDs in broadband optoelectronic devices such as optical limiters in the visible range or saturable absorbers in the near-IR range.
